# Influence of Fragment Size on the Time and Temperature of Ethylene Vinyl Acetate Lamination Decomposition in the Photovoltaic Module Recycling Process

**DOI:** 10.3390/ma12182857

**Published:** 2019-09-05

**Authors:** Anna Kuczyńska-Łażewska, Ewa Klugmann-Radziemska

**Affiliations:** Faculty of Chemistry, Gdansk University of Technology, ul. Narutowicza 11/12, PL-80-233 Gdansk, Poland

**Keywords:** EVA, photovoltaic, recycling

## Abstract

Photovoltaics is a commercially available and reliable technology with significant potential for long-term growth in nearly all global regions. Several research institutes and companies are working on recycling concepts for thin film modules and modules with crystalline cells. The establishment of recycling and reuse technologies appropriate and applicable to all photovoltaics (PV) modules is a key issue to be addressed as part of corporate social responsibility to safeguard the environment and to implement a fully material-circulated society without any waste. The copolymer ethylene-vinyl acetate (EVA) layer is a thermoplastic containing cross-linkable ethylene vinyl acetate, which is used to encapsulate the photovoltaic cells. The cells are laminated between films of EVA in a vacuum, under compression, and up to 150·°C. The encapsulant’s primary purpose is to bond or laminate the multiple layers of the module together. In the photovoltaic module recycling process, the second important step (after mechanical dismantling of the frame) is EVA lamination removal. In this study, different parameters of the thermal delamination method used during the recycling process were experimentally tested and compared, and the most ecological and economical one is proposed.

## 1. Introduction

Compared to other energy-producing techniques, photovoltaics (PV) are one of the most promising options: There is no emission of any matter into the environment during operation, they have extremely long operation periods, they require minimum maintenance, and it is a robust technique with aesthetic aspects.

PV modules contain various high-value materials that, in many cases, can be economically recovered. The procedure for recovering valuable constituent materials from monocrystalline, polycrystalline, and thin-film modules is as follows: removing and collecting the metal frame, junction box, and cable; separating the cover glass; removing the EVA (copolymer ethylene-vinyl acetate) from the surface of the devices, and chemical treatment of the parts.

The major factor controlling the lifetime of the module is the deterioration of the encapsulant resin by UV rays and the breakage of interconnecting wires by thermal stress. In the range of 40 to 1100 nm, encapsulant resin shows nearly the same optical transmission as glass. The properties that make EVA the encapsulation material of choice are high electrical resistivity, low fusion and polymerization temperature, low water absorption ratio, and good optical transmission. EVA can be removed by dissolution, thermal decomposition, and fluidized bed combustion [1].

In the past, the main focus of recycling efforts was the recovery of complete silicon crystalline cells, which were separated from the module using thermal decomposition of the plastic encapsulation. The recovered wafers were then reprocessed in an etching line and used for new module assemblies with no apparent loss in performance. However, as the thickness of the wafers decreases, it is expected to become more difficult to recover intact cells. Today, the main focus is to recover the silicon as raw material, recovering separate pure fractions of different metals, glass, and silicon [2].

In the case of silicon crystalline modules and the recovery of complete cells, the cell recovery rate, defined as the ratio of the number of cells removed without damage to the number of cells supplied in the PV module, is ~85%, regardless of the method [3].

The economic side of the recycling process is one of the strong functions. One the one hand, there is the concentration and value of reclaimable materials; on the other, there is the cost of reclamation. The greatest effort is in separating valuable materials from the rest of the module. Delamination is the main step in the evaluation of whether the recycling process will be economically profitable [4]. Greater effort is required in regard to changing the law in Europe [WEEE (Waste of Electrical and Electronic Equipment Directive 2012/19/EU) and RoHS (Restriction of Hazardous Substances Directive 2011/65/EU)]. This especially concerns thin-film module technology that contains heavy metals, such as cadmium, copper, selenium, and lead [5].

**Figure 1:**
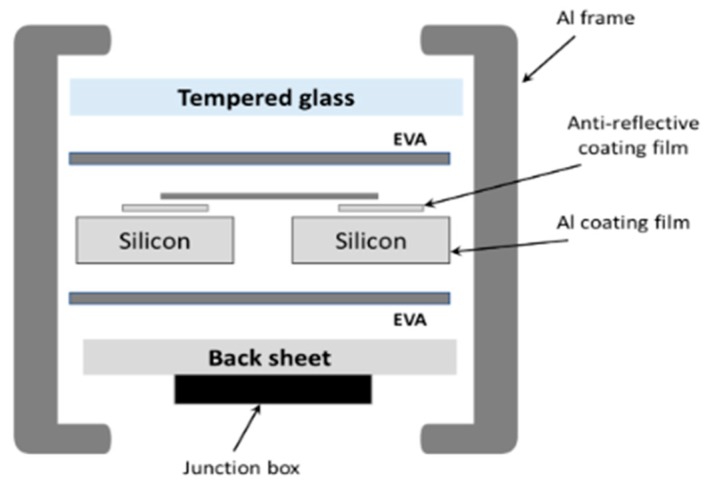
Cross-sectional view of the typical structure of the c–Si photovoltaic (PV) module.

Laminate mostly consists of EVA or Tedlar^®^ (polyvinyl fluoride). The crystalline silicon module consists of 10 wt.% polymer and more than 70 wt.% glass [6,7] (Figure 1).

Few delamination methods are used during the recycling process. The most popular have been described by Marwede et al., 2013 [8]. The easiest way is physical disintegration by milling whole modules [6,9] or cutting the layer of the laminate. The efficiency of this method is low because the EVA foil only partially peels off the glass and it is hard to fully separate the semiconductor from the glass. The microemulsion method is one of the newest methods, as well as crushing at the temperature of liquid nitrogen, e.g., −196 °C, but this is a very expensive method of delamination. In the late 1990s [10], delamination was connected to dissolving the semiconductor layer, for example, with 8 N H_2_SO_4_ and 1% H_2_O_2_.

The most common ways are thermal decomposition of the organic foil or dissolving the EVA polymer in organic solvents (Table 1) [7,11]. Best results were obtained at 80 °C during a 10 min treatment. For room temperature and 2 days treatment, trichloroethylene and tetrahydrofurane were able to dissolve the EVA foil. Both methods are potentially harmful to the environment [2]. There are many different temperatures (300–600 °C) and parameters (under air or Ar) of EVA polymer pyrolysis [7,12,13,14]. During thermal treatment above 450 °C or treatment with inorganic acids, cells can become defective. Most organic solvents cause swelling of the EVA foil and, therefore, cause cell breaking. The process of dissolution can be accelerated by ultrasonic irradiation (900 W irradiation), which shortens the time of delamination from seven days to 30 min and protects the cells from cracking [15].

The experimental purpose of this paper is to find the optimal temperature and fragment size for the delamination process. These experiments are very important to the recycling process of PV modules because delamination is the key step in the process. Gridding which is connected with a higher cost in electric energy consumption, but smaller fragment size, and has influence on the shorter time and lower temperature of delamination. This research aimed to find optimal parameters for thermal delamination.

## 2. Material Description

The main role of the lamination material is to bond or laminate the multiple layers of a module together.

A good encapsulant should have properties such as high optical transmittance, strong adhesion to different materials, suitable mechanical compliance to accommodate stresses, and good dielectric properties [16]. A variety of lamination materials have been used, but the most popular is EVA in different formulations. In the photovoltaic module, the EVA interlayer film ensures high performance of photovoltaic panels by withstanding the harmful effects of ultraviolet rays and severe weather, simultaneously allowing broad band light transmission to solar cells.

**Figure 2:**
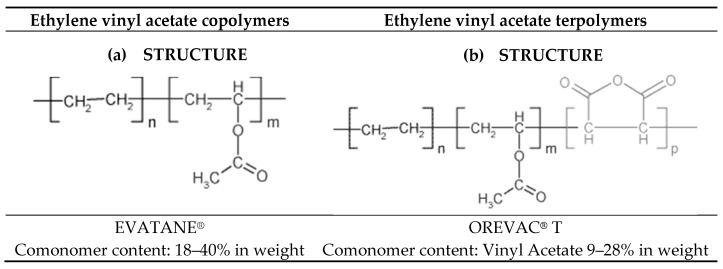
Structures of two kinds of EVA polymers (**a**) Ethylene vinyl acetate copolymers, (**b**) Ethylene vinyl acetate terpolymers.

The properties of the EVA copolymer depend on the percent composition of ethylene and vinyl acetate or the addition of UV absorbers and photo- and thermo-antioxidants [17]. Depending on the composition of the copolymer (Figure 2), EVA decomposes at around 350 °C [13,18].

An overview of materials for EVA copolymers can be found for similar materials in the database [19]. Each property range of values reported is the minimum and maximum values of appropriate entries. The comments report the average value and the number of data points used to calculate the average. The values are not necessarily typical of any specific grade, especially less common values and those that are most affected by additives or processing methods.

For the DSC (differential scanning calorimetry) measurement starting at a temperature below room temperature (−23–227 °C), the non-cross-linked sample had two peaks at 50 °C and 70 °C. An exothermic double peak appears in the range of 140 to 200 °C. For the cross-linked sample, the exothermic peak is missing, and the sample had a broad endothermic shoulder (20–50 °C) without any peak. The first peak appears at 65 °C [20].

**Figure 3:**
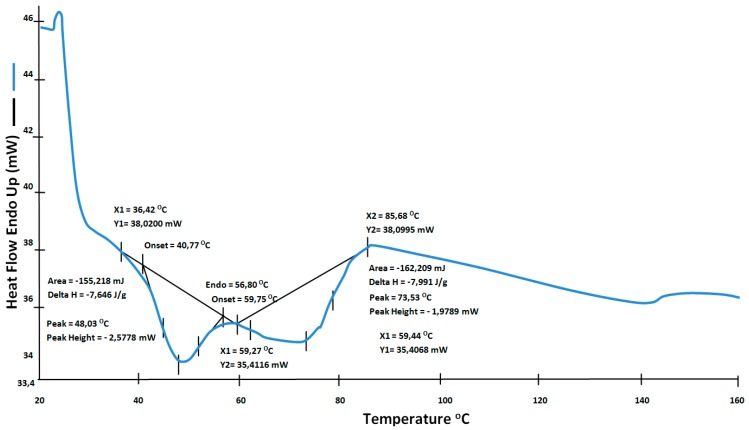
Thermal analysis of the ethylene-vinyl acetate (EVA) probe.

Figure 3 presents the DSC results. There are two peaks clearly visible: one for a temperature of about 50 °C and the second for a temperature higher than 70 °C. The first peak cannot be the melting point of the EVA; this temperature is too low and is associated with crystallization. Therefore, the melting temperature of the copolymer is the value that was recorded for the exothermic peak at 85.68 °C. Additionally, samples cut from the EVA film were placed in a Boëtius microscope and were gradually heated to a temperature of 220 °C; the changes were observed over time. The obtained visual changes in the structure of EVA allowed us to determine the melting point, which was about 80 to 90 °C for the tested EVA copolymer [21].

## 3. Experimental

### 3.1. Materials

Two types of samples were examined: samples of the pure EVA film, produced for photovoltaics, and the EVA laminated PV module, which was prepared by cutting.

The EVA used in the experiments has extremely high transparency; outstanding heat, humidity, and ultraviolet ray durability; and long-term reliability. It can be stored at room temperature and processed using low-cost equipment, is highly adhesive to materials other than glass, and has excellent sound insulating properties in the high-frequency range.

EVA foil materials used in the experiments were produced by several companies, such as Hangzhou First PV Material Co. (Hangzhou, China) (3), Bridgestone Corporation (Tokyo, Japan) (4), NovoPolymer (Puurs, Belgium) (5), Hanwha (Seoul, South Korea) (6), EVA-SA (A Coruña, Spain) (7), Changzhou Sveck PV New Material Co. (Changzhou, China) (8), and sample from Photovoltaic Laboratory of Polish Academy of Sciences in Kozy, Poland (1). The sample properties from the producers’ websites are presented in Table 2. The measured thickness of all samples was between 0.4 and 0.5 mm to assure reproducibility of the experiment.

PV module pieces cut from a thin film CdTe module produced by Advanced Solar Power Hangzhou INC (Hangzhou, China) made up the second type of samples. Pieces were cut from the center of the module without bus bars. Module pieces were milled in a Retsch (Haan, Germany) PM100 planetary mill with a zirconium oxide bowl and eight balls (30 mm).

At the beginning of the experiment, thermal analysis of the eight samples of EVA foil was performed by DSC (Q20 DSC, TA Instrument, New Castle, DE, USA). All thermal analysis experiments were carried out at a heating rate of 10 °C/min in the temperature range of −25 to 225 °C in four cycles.

### 3.2. Thermal Treatment

For thermal treatment, the following samples were prepared: 5 cm × 5 cm (40 g), 3 cm × 3 cm (15 g), and 1 cm × 1 cm (1.5 g) pieces; 4 g of powder (grains under 250 µm) of the thin film CdTe module; and 4 g of EVA foil pieces were placed in a crucible. The samples were heated in a horizontal pipe furnace (PTF 12/105/500, Protherm, Ankara, Turkey) at different temperatures (300–800 °C) with air access for 1 h, 3 h, and 5 h. The heating rate was 10 °C/min. The time was measured from the moment the temperature was determined. Afterwards, the sample was weighed and the weight loss was recorded.

## 4. Results and Discussion

### 4.1. Material Characteristics

**Figure 4:**
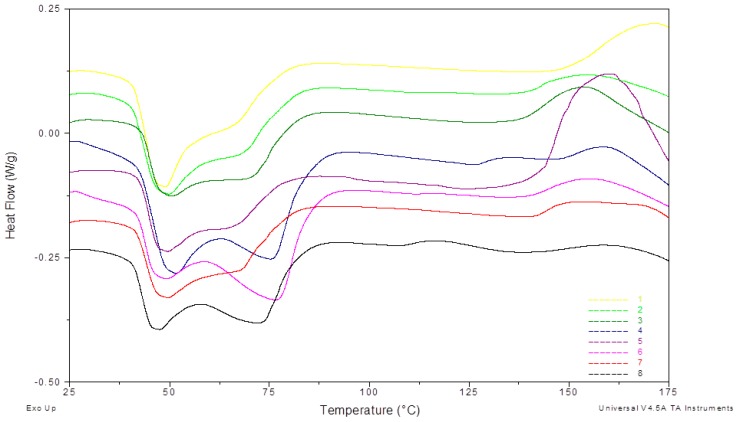
Differential scanning calorimetry (DSC)—heat flow results from the first cycle.

The DSC measurement results for EVA foil samples are shown in Figure 4 which shows the results for samples from eight different producers for the heating run. Between 40 and 90 °C, two endothermic peaks appear. Depending on the producer, the peaks are more broad or distinct. According to [20], the broad endothermic shoulder is specific for cross-linked materials. This means that temperature treatment above the cross-linking temperature permanently changes the crystalline structure.

The following peak appears above 130 °C and stands out only for a few producers. According to [20], the exothermic peak represents the cross-linking reaction in the polymer.

This means that the samples with lower cross-linking degrees will have more distinct peaks, and those with higher degrees will have a broader peak or no peak at all.

The DSC measurement results show that producers use EVA foils with different parameters in PV modules’ lamination process. Prepared samples have different cross-linking degrees and can be a reliable source of information as a representative for foils used in the industry. Experiments performed on such a prepared group of samples give a wide spectrum of effects that can occur during the recycling process. For example, foil swelling process while dissolving in solvents causes glass cracking or different parameters of thermal decomposition.

### 4.2. Thermal Treatment

**Figure 5:**
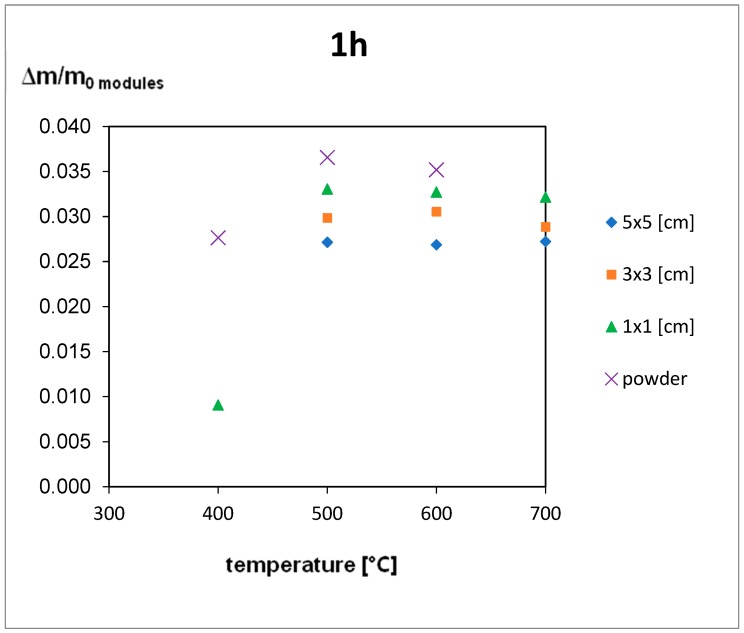
Results of the thermal treatment of module pieces for 1 h.

**Figure 6:**
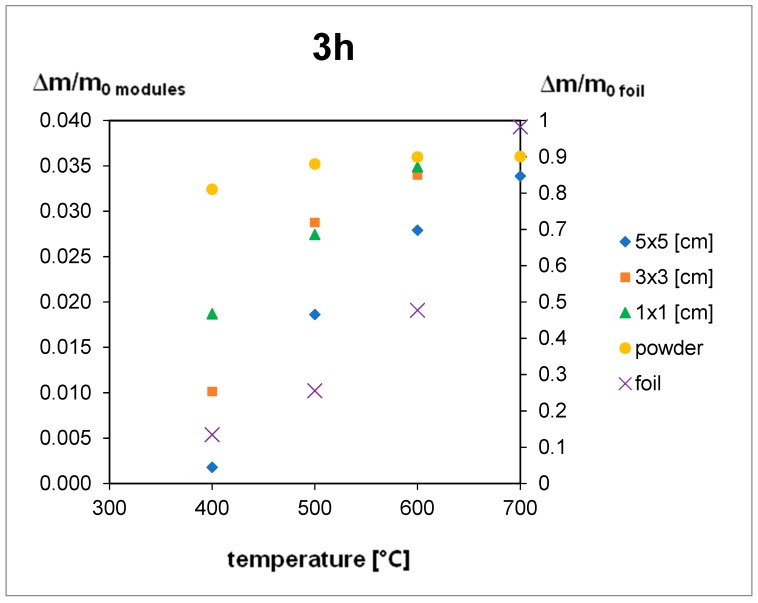
Results of the thermal treatment of module pieces and EVA foil for 3 h.

**Figure 7:**
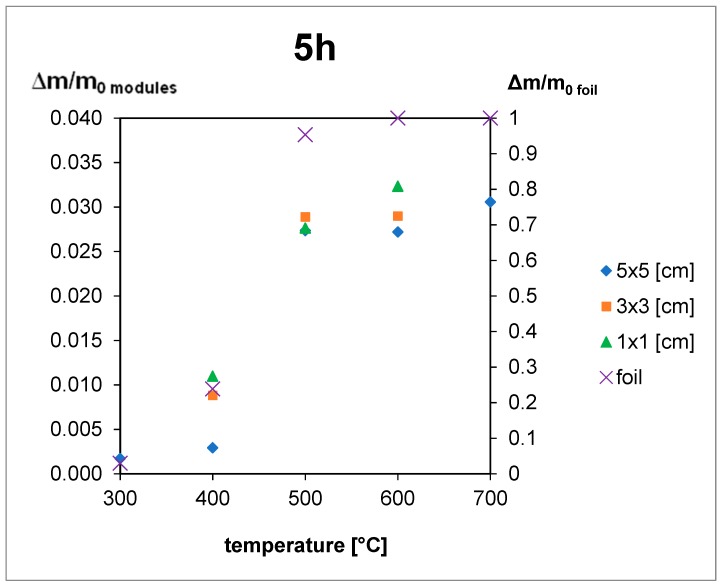
Results of the thermal treatment of module pieces and EVA foil for 5 h.

The thermal treatment results are shown in Figure 5, Figure 6 and Figure 7. For the 5 h thermal treatment, the size of the pieces has no influence on the mass loss. For 3 and 1 h treatments, sample size influences at 400 °C can be seen. In every case, the minimum temperature to obtain total EVA degradation is 500 °C, but for powder gradation (<250 µm), a very high mass loss can be seen, even for 400 °C.

The EVA foil samples were thermally decomposed after 3 h at a temperature of 700 °C and after 5 h at 600 °C. For the samples of the module pieces, despite the decomposition time, the temperature had to reach 500 °C to complete the decomposition of EVA. This means that during the module treatment the increase of temperature to 500 °C is obligatory, and time elongation is not efficient (Figure 5, Figure 6 and Figure 7), but for powder gradation, the temperature can be decreased below 500 °C for energy savings.

Extending time for the powdered module samples was not needed as 3 h treatment in 400 °C was efficient. Results for pure foil degradation after 3 h treatment were not sufficient below 700 °C so shorter time would be unproductive.

Results for 5 × 5 cm pieces after 1 h treatment can contain a bigger error than other samples, especially between 400 and 600 °C. In this temperature range, EVA foil starts to form a glass-like structure which it is very hard to remove from the crucible after a shorter treatment so there can be a weight loss error. This might be the reason why for the 3 h treatment weight loss is smaller than the 1 h treatment at the same temperature of 500 °C.

**Figure 8:**
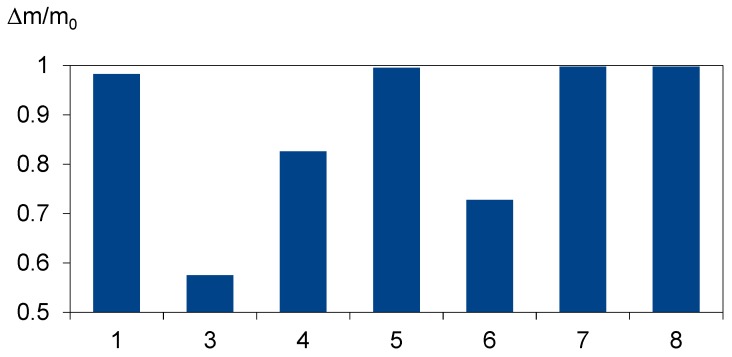
Results of the thermal treatment of EVA foil from different producers at 700 °C for 3 h.

The results presented in Figure 8 show that the degree of foil decomposition depends on the properties of the foil. It can be assumed that the main features are the cross-linking degree and vinyl acetate content percentage. Three kinds of foil have an unsatisfactory degree of decomposition. The samples were carbonized, but some foil residues stayed in the crucible. The smaller degree of decomposition does not mean that glass sheets cannot be separated in these conditions. From the recycling process perspective, it would be better if the manufacturer provided information on what kind of foil was used or to implement uniform parameters for EVA foil.

During thermal decomposition, the evolution of harmful gases can occur. The temperature for polyethylene thermal decomposition is 490 to 506 °C in a nitrogen atmosphere, but the presence of oxygen enhances the process, and weight loss is already detected at 150 °C [22]. Without oxygen, many hydrocarbons can be released into the atmosphere, such as propane, propene, ethane, ethene, butene, hexene-1, and butene-1. In an air atmosphere, oxidation processes provide a total decomposition of harmful substances above 500 °C. For poly (vinyl acetate) aromatization by hydrogen evolution occurs at roughly 450 °C without oxygen after acetic acid evolution. Above 430 °C with oxygen access, char (generated after acetic acid evolution) is oxidized without residue [22]. This is the reason why experiments were conducted in air conditions with full oxygen access to ensure total decomposition.

Results presented above help to choose the best size for pieces used in the recycling process. The best results were achieved for powder samples, but the cost of milling can influence the profitability of the whole process. An important finding is that for bigger pieces (5 × 5 cm), time elongation is not efficient and higher temperatures are needed. Authors would suggest that the optimal process would be 3 × 3 cm size pieces at 600 °C for 1 h treatment according to the presented results. Short time and relatively low temperature and big size of pieces will optimize the energy input.

## 5. Conclusions

The idea of reusing materials to create a society without any waste is a noble goal. The key point is to be aware that manufacturers are obligated to take part in the recycling process of end-life PV modules. It is important to remember that recycling technology should also be environmentally friendly. Delamination is one of the main problems in recycling PV modules.

DSC measurements confirmed that the prepared group of samples had different physical and chemical properties, which can be a reliable source of different effects that occur during the recycling process.

According to the experimental results, it is obligatory to increase the temperature to a minimum of 500 °C during thermal PV module treatment. Time elongation is not efficient for big pieces (5 cm × 5 cm), but decreasing the size of the pieces shortens the processing time. The properties of the foil play a main role in the degree of foil decomposition; the main features are cross-linking degree and the percentage of vinyl acetate content.

The next step of the experiment will be the inspection of the environmental impact of the presented method. During thermal treatment, harmful compounds can be released without enough oxygen access. A system controlling the air amount and waste gases should be applied.

## Figures and Tables

**Table 1:** Effects of organic solvents on the physical properties of the ethylene-vinyl acetate (EVA) polymer.

Solvent	Room Temperature/2 Days	80 °C/10 min
**Acetone**	n	DS
**Toluene**	SW	DS
**Petroleum benzine**	n/SW	DS
**Isopropanol**	n	DS
**Methyl ethyl/isobutyl ketone**	n/SW	DS
**Tetrahydrofuran**	SW/DS	DS
**Ethylene glycol**	n	n
**Trichloroethylene**	SW/DS	DS
**Glycerine**	n	n
**Ethyl alcohol**	n	-

n—no change, SW—foil swelling, DS—foil dissolution, -—no data.

**Table 2:** Physical, chemical, and optical properties of EVA samples used in the experiment.

SAMPLE NUMBER	AVERAGE THICKNESS [mm]	DENSITY [g/cm^3^]	LIGHT TRANSMISSION [%]	CROSSLINKING DEGREE [%]	VA CONTENT [wt%]
1	0.4	nd	Nd	nd	nd
3	0.3–0.8 (measured 0.4–0.5)	nd	>91	>75	28–32
4	0.4	nd	>87	nd	nd
5	0.2–1.1 (measured 0.4–0.5)	0.95	89	nd	nd
6	Nd	0.94	Nd	nd	19–22
7	0.46	0.96	91	>85	nd
8	0.45–0.5	0.96	>92	>75	nd
